# Increased Intestinal Permeability and Stool Zonulin, Calprotectin and Beta-Defensin-2 Concentrations in Allogenic Hematopoietic Cell Transplantation Recipients

**DOI:** 10.3390/ijms232415962

**Published:** 2022-12-15

**Authors:** Martyna Tyszka, Dominika Maciejewska-Markiewicz, Jarosław Biliński, Arkadiusz Lubas, Ewa Stachowska, Grzegorz W. Basak

**Affiliations:** 1 Department of Hematology, Transplantation and Internal Medicine, Medical University of Warsaw, 02-091 Warsaw, Poland; 2 Department of Human Nutrition and Metabolomics, Pomeranian Medical University, 71-460 Szczecin, Poland; 3 Department of Internal Medicine, Nephrology and Dialysis, Military Institute of Medicine, 04-141 Warsaw, Poland

**Keywords:** intestinal barrier, gut permeability, allogeneic hematopoietic cell transplantation, graft-versus-host disease

## Abstract

Significant progress has been made in understanding the connection between intestinal barrier function and allogenic hematopoietic cell transplantation (allo-HCT) recipients’ outcomes. The purpose of this study was to further evaluate gut barrier permeability and other potential intestinal barrier disruption markers in the allo-HCT setting. Fifty-one patients were enrolled in the study. Intestinal permeability was assessed with the sugar absorption test and faecal concentrations of the zonulin, calprotectin and beta-defensin-2 levels in the peri-transplantation period. Most patients undergoing allo-HCT in our department had a disrupted intestinal barrier at the baseline, which was associated with older age and higher Hematopoietic Cell Transplantation-specific Comorbidity Index (HCT-CI). Regardless of this, we observed a further increase in gut barrier permeability after allo-HCT in most patients. However, there was no association between permeability assay and other markers (zonulin, calprotectin and beta-defensin-2). Patients with acute GVHD had significantly higher median calprotectin concentrations after allo-HCT compared with the patients without this complication. Our findings indicate that gut barrier damage develops prior to allo-HCT with progression after the procedure and precedes further complications, but did not prove other markers to be useful surrogates of intestinal permeability.

## 1. Introduction

Allogenic hematopoietic cell transplantation (allo-HCT) remains the only curative option for many hematological malignancies and other diseases, but its application is limited due to the risk of life-threatening complications, mainly graft-versus-host disease (GVHD) and infections. In the past few decades, there has been a tremendous growth in interest regarding the connection between intestinal barrier function and allo-HCT recipients’ outcomes [1,2,3,4,5,6,7,8]. The intestinal epithelial barrier is a complex molecular communication machinery that allows nutrient absorption and the selective transfer of various molecules across the intestinal wall and protects against harmful toxins translocation into the body fluids. It is regulated by a complicated interplay between epithelial cells, intestinal microbiota, the immune system and the enteric nervous system [9].

Gastrointestinal toxicity is carefully observed in the posttransplant clinical setting. Practically oral mucositis was evaluated with a World Health Organization (WHO) score [10]. Intestinal permeability after allo-HCT is only a subject of clinical research. It is most commonly measured as a ratio between urinary excretion of various ingested probes compared to the control—mainly with the 51CrEDTA absorption test or sugar absorption test (SAT). Few studies evaluating intestinal barrier function after allo-HCT have documented that gut permeability increases significantly after transplantation. It was also observed that the peri-transplant increase in intestinal permeability anticipates the occurrence of mucositis but does not strictly correspond with it. Hence, it may be misleading to estimate the intestinal injury on the extent of oral mucositis in the same patient [11,12,13,14].

**Figure 1:**
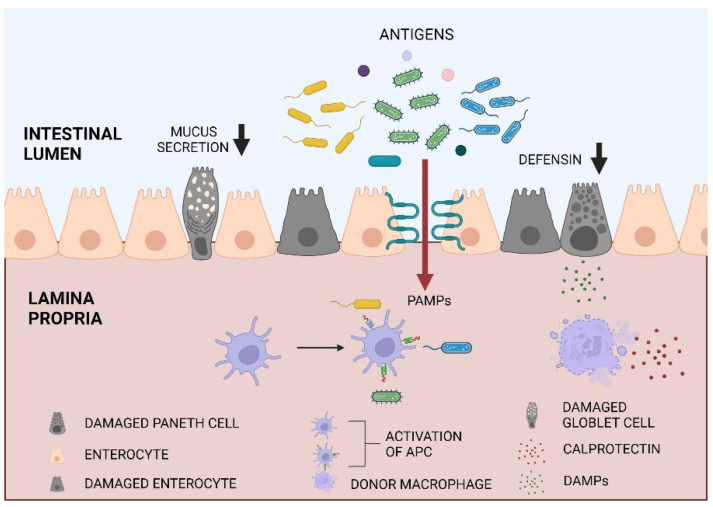
Intestinal barrier disruption after allo-HCT. Pre-transplant conditioning results in enterocyte, goblet cells and Paneth cells apoptosis. Increased MLCK expression results in loosening of the tight junctions and PAMPS translocation into the lamina propria. PAMPS (e.g., LPS) and DAMPs from damaged cells activate APCs. It results in donor T cell activation, pro-inflammatory cytokines i.e., IFN-γ, TNF-α, IL-5 release and GVHD initiation. This figure was created with BioRender.

Most evidence on intestinal barrier importance in the allo-HCT setting was seen in the GVHD development [3,4,5,6]. Conditioning chemotherapy leads to the disruption of the intestinal barrier and increases its permeability. It allows pathogen-associated molecular patterns (PAMPs) translocation to the lamina propria and faulty activation of the gut’s wall immune system, promoting GVHD initiation [15], as shown schematically in Figure 1. A study assessing intestinal permeability after allo-HCT with 51Cr-EDTA absorption test confirmed that patients with preserved intestinal barrier function (‘normal’ permeability) experienced less gut toxicity and mild acute GVHD (aGVHD) compared to those with increased permeability [14]. The authors stressed that among other characteristics related to the patient or transplant procedure, the intestinal barrier permeability and intestinal toxicity were the only factors significantly correlating with the severity of aGVHD [14]. In another study, it was proven that a proper intestinal barrier expressed as preserved microbial diversity was associated with significantly lower mortality after allo-HCT [16]. 

MLCK; myosin light chain kinase, PAMPs; pathogen-associated molecular patterns, LPS; lipopolysaccharide, DAMPs; damage-associated molecular patterns, APC; antigen-presenting cell, IFN-γ; interferon gamma, TNF-α; tumor necrosis factor alpha, IL-5; interleukin 5, GVHD; graft-versus-host disease.

Other tools for intestinal barrier disruption and increased permeability evaluation are biomarkers assessed in urine, serum or stool. Those particles can be divided into bacteria-related molecules such as lipopolysaccharide (LPS) [17] and circulating endotoxin core antibodies (EndoCAb) [18], or direct intestinal barrier damage markers such as citrulline produced by small intestinal enterocytes [19,20], fatty acid-binding proteins (FABPs) [21], tight junctions proteins—claudins [22] or calprotectin—a sensitive marker for mucosal inflammation of the intestine [23]. It was recently observed that in patients with confirmed gastrointestinal aGVHD, the density of donor-calprotectin-expressing colonic mucosal macrophages was significantly increased compared to the patients without aGVHD, again stressing that inflammation at the level of the intestinal wall is strictly associated with aGVHD development [24]. Zonulin is another recently discovered intestinal permeability marker. It is believed to act in a similar mode to the Zonula occludens toxin produced by Vibrio cholerae, relaxing enterocytes’ tight junctions (TJs) and increasing paracellular transport via the intestinal wall [25,26]. Zonulin is overexpressed in autoimmune diseases where TJ dysfunction plays a pivotal role, such as celiac disease and type 1 diabetes [25]. Since the discovery of zonulin by Fasano in 2000, this protein has been used as a biomarker of several immune-mediated diseases, including autoimmune diseases, malignancies and neuro-inflammatory diseases [27]. Another peptide involved in gut barrier preservation is beta-defensin-2, which is believed to promote cell proliferation, anti-inflammatory responses and intestinal barrier’s TJs formation and restoration [28,29].

This study aimed to test the relationship between intestinal permeability assessed with a well-established sugar absorption test and zonulin, calprotectin and beta-defensin-2 concentrations in patients before and during the post-transplant period. We also attempted to correlate the zonulin, calprotectin and beta-defensin-2 levels and intestinal permeability with a set of patients and transplant characteristics and transplantation complications.

## 2. Results

### 2.1. Increased Intestinal Permeability in Allo-HCT Recipients

The median lactulose to mannitol ratio in the urine (LMR) before the conditioning was 0.04 (0.002–1.084), which is above the reference range for the whole group of 45 patients that underwent a sugar absorption test (SAT). 

Twenty four patients (53%) already had elevated LMR at the baseline before conditioning regimen initiation with a median value of 0.190 (0.039–1.084), while in the remaining 21 (47%) it was within the normal range, with a median equal to 0.028 (0.002–0.034). There was a significant correlation between LMR at −7 and HCT CI score (lower LMR in the group with HCT CI of 0–2 and higher for the group with HCT-CI > 2 on day −7 (median 0.041; 0.002–1.084 vs. 0.43; 0.03–1.03, *p* = 0.035).

**Figure 2:**
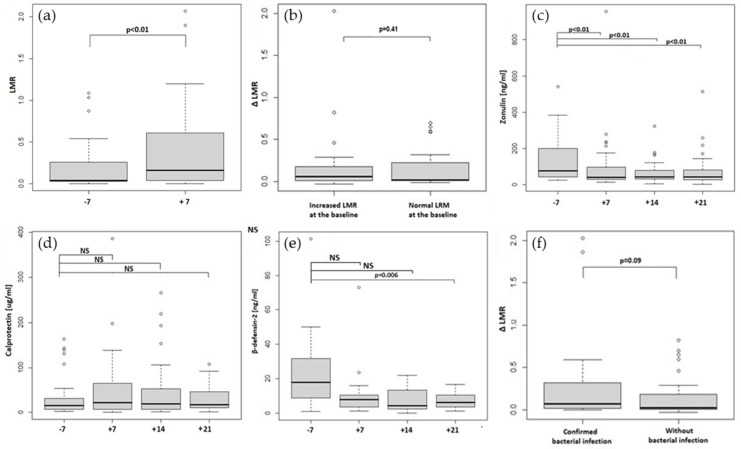
(**a**) increased intestinal permeability before and after the allo-HCT expressed as lactulose to mannitol ratio in the urine after the SAT test for the whole group; (**b**) change in intestinal permeability (ΔLMR) in patients with already increased LMR at the baseline and patients with LMR at the baseline within the reference limit; (**c**) zonulin stool concentrations before the conditioning and in the post-transplant period; (**d**) calprotectin stool concentrations before the conditioning and in the post-transplant period; (**e**) beta-defensin-2 stool concentrations before the conditioning and in the post-transplant period; (**f**) change in intestinal permeability (ΔLMR) in patients with confirmed bacterial infection compared to those without this complication. A *p*-value less than 0.05 is considered to be statistically significant.

Thirty four out of 45 patients (76%) had intestinal permeability increased on day +7 (median 0.242; 0.03–2.068) and median LMR for the whole group on day +7 was 0.16; 0.002–2.068, *p* < 0.05, as shown in Figure 2a. Interestingly, there was a remarkable, but not statistical difference in day +7 (but not day −7) LMR between patients < and >65 yo (median 0.108; 0.02–2.068 vs. median 0.7 (0.03–1.1), as shown in Table 1. Interestingly, in the group with a worse transplant risk (HCT CI > 2), the ΔLMR was higher (0.09; −0.014–0.290) than in patients in the better risk group (HCT CI ≤ 2; 0.036; −0.03–2.027) *p* = 0.903, and a similar correlation was no longer observed in the age groups (Table 1). We did not observe significant correlations between median LMR on day −7 or day +7 and other patient or transplantation characteristics (Table 1).

Patients with increased permeability before transplantation seemed to experience a more considerable rise in LMR expressed as ΔLMR (median ΔLMR 0.060; −0.030–2.027) compared to patients with a normal ratio before transplantation (median ΔLMR 0.016; −0.014–0696; *p* > 0.05, as shown in Figure 2b. 

### 2.2. Zonulin, Calprotectin and Beta-Defensin-2 Stool Concentrations

Zonulin, calprotectin and beta-defensin-2 stool concentrations were assessed on day −7, +7, +14 and +21 after transplantation. Zonulin levels were significantly increased before the conditioning regimen initiation (76.45 ng/mL; 23.45–2040.35) and at the following time points compared to laboratory normal values < 30 ng/mL. In addition, we observed a significant decrease in the median zonulin level on day +7 after the conditioning (median 39.95 ng/mL; range;12.75–955.60 with *p* = 0.001), which remained significantly decreased until day +21 (Table 2 and Figure 2c).

Median calprotectin levels were within the normal range at the baseline and during the post-transplantation period, not significantly rising after the conditioning, as shown in Table 2 and Figure 2d.

Beta-defensin-2 median levels were also within the normal range during the transplantation period. However, we have seen a significant drop in beta-defensin-2 concentrations on day +21, as shown in Table 2 and Figure 2e. 

### 2.3. Intestinal Permeability and Stool Biomarker Associations

There were no significant relationships between intestinal permeability expressed with LMR or ΔLMR and stool concentrations of zonulin, calprotectin or beta-defensin-2 as assessed with a Spearman’s rank correlation test.

### 2.4. Correlations between the Transplantation Complications, Intestinal Permeability and Stool Biomarkers

#### 2.4.1. Infectious Complications

Among all patients that underwent SAT, 14 had bacterial infection with positive culture within 30 days post-allo-HCT (confirmed bacterial infection). These patients had a higher, but not significantly median ΔLMR of 0.069 (−0.001–2.027) compared with a median ΔLMR of 0.020 (−0.030–0.820) in those who did not experience proven infection (Figure 2f). Furthermore, we did not see any relationship between stool biomarker levels and the incidence of a bacterial infection within 30 days after allo-HCT (as shown in Supplemental Table S1).

#### 2.4.2. aGVHD

Among all patients, 16 developed aGVHD of any grade within 30 days of the transplantation (cutaneus in 11, hepatic in two, intestinal in three). We obtained the sample for calprotectin evaluation in 6 of the patients (5 with skin aGVHD and 1 with intestinal aGVHD). This group had higher calprotectin levels assessed on day 7 after the conditioning regimen compared to the group of patients without aGVHD (64.8 ng/mL vs. 14.1 ng/mL, *p* = 0.044) as shown in Table 3. Surprisingly, patients who developed aGVHD had a lower concentrations of zonulin in the stool samples before the conditioning and across the whole transplantation period time-points comparing to patients without aGVHD (however the difference was not significant). There were no other significant differences in intestinal permeability or biomarkers between groups with and without aGVHD (as shown in Supplemental Table S2). 

#### 2.4.3. Mucositis

We have not observed a correlation between the risk of developing mucositis of any grade or of grade III and IV in patients with increased intestinal permeability expressed as LMR or stool biomarkers correlation as shown in Supplemental Table S3. In our study, mucositis development was only associated with myeloablative conditioning (*p* = 0.031).

## 3. Discussion

In this study we wanted to assess the potential relationship between intestinal permeability assessed with a well-established sugar absorption test and certain markers of intestinal barrier damage i.e., zonulin, calprotectin and beta-defensin-2 concentrations in patients before and during the post-transplant period. We also decided to investigate the correlation between zonulin, calprotectin and beta-defensin-2 levels and intestinal permeability with patients and transplant characteristics and transplantation complications. The main limitations of this study were the small number of patients that agreed to participate and problems with compliance regarding the stool collection. This issue led to the further decrease in the number of samples needed for biomarkers analysis.

Most patients undergoing allo-HCT had disrupted intestinal barriers and increased intestinal permeability measured as LMR after SAT before the start of the conditioning therapy. This was probably due to antibiotics use, damage to the gut by the cytotoxic chemotherapy, or leukemic infiltrations [30]. These findings are in line with previously published work. In the study by Sundström et al., intestinal permeability measured with SAT was significantly increased in 16 adult patients with de novo AML in comparison with healthy controls before, during and after induction chemotherapy [31]. Similar results were obtained by Blijlevens et al. when SAT was performed in a group of 18 patients undergoing cytotoxic chemotherapy for newly diagnosed AML or MDS [13]. Another small study on 20 leukemic patients has shown a trend toward increased intestinal permeability before the chemotherapy compared to healthy volunteers [32]. SAT is the reference test to assess intestinal permeability but has its limitations, i.e., it cannot distinguish between increased tight junction permeability and epithelial cell damage. Other disadvantages are the troublesome need for 6 h urine collection and the risk of diarrhea induction. Because of this, SAT did not become useful in clinical practice. In our research, we wanted to explore other potential intestinal permeability biomarkers for allo-HCT recipients. In recent years, zonulin has become one of the most popular tests for assessing leaky gut in many conditions [25,26,27]. However, it seems that zonulin release is not always the major mechanism responsible for the increase in intestinal permeability. To the best of our knowledge this was the first attempt to evaluate zonulin concentrations in the allo-HCT setting; however, our findings did not show any association between the permeability assay and zonulin concentrations. Surprisingly, zonulin concentration was elevated before the start of the conditioning in a majority of the patients (88%) and dropped significantly on day +7 after the HCT. In the subgroup of the patients who developed aGVHD, zonulin concentrations were lower than those without aGVHD, although this was not statistically significant. So far, these results are difficult to interpret. One possible explanation is that intensive conditioning before allo-HCT leads to the destruction of enterocytes and therefore impedes zonulin release. Zonulin’s role in the development of intestinal barrier disruption and aGVHD in the allo-HCT setting needs more evaluation, possibly by evaluating and comparing serum and faecal zonulin concentrations before and after the conditioning.

Fecal beta-defensin-2 is an established intestinal inflammation marker that was used in several clinical trials mainly in the pediatric population to asses to effect of dietary interventions on infant colic [33], intestinal barrier permeability [34], and the occurrence of atopy [35] and infectious diseases [36]. To the best of our knowledge, beta-defensin-2 was not previously assessed in the allo-HCT patients. In our study, beta-defensin-2 levels decreased in the post-transplant period, although it was not statistically significant (probably due to the very small sample size for this evaluation). Data from in vitro and animal model studies [27,28] shows a beta-defensin-2 protective function during intestinal barrier damage, so it might be assumed that the lack of beta-defensin-2, possibly due to the damage of the intestinal epithelium, adds to the further gut barrier disruption in our patients. However, this assumption needs further evaluation.

A total of 58% of our patients experienced infectious complications within the first 30 days after allo-HCT, and 29% had a bacterial infection confirmed by a positive blood or urine culture. In addition, we had seen a trend showing that the risk of bacterial complications was augmented when patients had a higher increase in intestinal permeability (as ΔLMR) which would confirm the results from previous studies on intestinal barrier integrity after allo-HCT, showing that decreased microbiota diversity in the peritransplant period increased the risk of bacteriemia [2], pulmonary infiltration [7], and transplant related mortality after allo-HCT (mainly aGVHD and infections) [8].

An aGVHD diagnosis is based on clinical symptoms confirmed by histopathological evaluation. However, the diagnosis could be problematic because GVHD symptoms are similar to other common transplantation complications such as infections or drug toxicity. Therefore, in recent years, there has been a tremendous interest in searching for GVHD biomarkers that could facilitate the diagnosis. One of the potential markers is faecal calprotectin. In our study, 16 patients were diagnosed with aGVHD (cutaneus in 11, hepatic in two, and intestinal in three). Calprotectin concentrations for the whole group of patients after allo-HCT were within the normal range along the transplantation period, but the aGVHD subgroup of patients in which calprotectin was evaluated (n = 6) had significantly higher median calprotectin concentrations at day +7 compared with the patients without this complication. This is in accordance with the latest metanalysis of 10 studies involving 494 patients, which showed an increase in the median calprotectin level in patients with GI-GVHD compared to non-GI-GVHD patients [37].

In our study, 71% of patients developed mucositis, with 43% of grades III and IV. Mucositis was associated with myeloablative conditioning, a recognized risk factor for mucositis development [38,39] but not with intestinal permeability expressed as LMR and stool biomarkers. 

Most of our patients had a disrupted intestinal barrier before the conditioning, which was associated with older age and higher HCT-CI. Increased intestinal barrier permeability assessed as LMR on day −7 preceded further gut barrier disruption expressed as ΔLMR and on subsequent days occurrence of bacterial infections. In our study, zonulin, calprotectin and beta-defensin-2 concentrations did not correlate with intestinal permeability expressed as LMR. This data is mostly preliminary and the role of faecal markers in the assessment of intestinal barrier permeability needs further investigation.

## 4. Materials and Methods

### 4.1. Patients

Fifty-one patients undergoing allo-HCT at the Department of Hematology, Transplantation and Internal Medicine, University Clinical Center, Medical University of Warsaw, Poland, who consented to participate in the study and sample collection was feasible were enrolled in the study between September 2017 and December 2021. Thirty-seven underwent myeloablative conditioning, and 14 underwent reduced intensity conditioning (RIC). Prophylaxis against GVHD among myeloablatively conditioned patients consisted of cyclosporine A (CsA) and four doses of methotrexate (MTX) (n = 28), CsA switched to tacrolimus (TAC) (n = 2), TAC, mycophenolate mofetil (MMF) and cyclophosphamide (n = 2). Anti-thymocyte globulin (ATG; 5 mg/kg/day for 2 consecutive days) was given when stem cells from an unrelated donor were used, together with CsA, MTX followed by TAC (n = 2), CsA and MMF (n = 1), TAC and MTX (n = 1) and TAC with MMF in one patient. 

In RIC patients, CsA with a course of MTX (n = 11) or CsA together with MMF (n = 2) and TAC with MMF (n = 1) were used.

Further details regarding patient characteristics and the allo-HCT procedure are given in Table 4.

### 4.2. Methods

Before starting the conditioning regimen (day −7), a differential sugar absorption test (SAT) with mannitol and lactulose was performed, and a urine sample was taken to determine the lactulose to mannitol ratio. SAT and urine collection were repeated on day +7. Stool samples were taken for the zonulin, calprotectin and beta-defensin-2 level determination on days −7, +7, +14 and +21 after allo-HCT. Faecal and urine samples were stored until the analysis at −80 °C. The material was transported under suitable conditions to the analysis site at the Department of Biochemistry and Human Nutrition of Pomeranian Medical University in Szczecin. 

#### 4.2.1. Sugar Absorption Test

Forty five patients participated in the SAT part of the study, while six patients did not agree to participate or met exclusion criteria for SAT. The test depends on the difference in pathways of gut absorption for lactulose and mannitol [40]. If intercellular tight junctions are damaged or relaxed, urinary excretion of lactulose, which is absorbed primarily through a paracellular pathway, increases relative to mannitol, which is transcellularly absorbed. Therefore, the differential sugar absorption test can be regarded as a direct marker of intestinal permeability. The test began in the morning and was preceded by an 8 h fast. First, each participant was asked to empty their bladder and subsequently drink 500 mL of a water solution containing 7.5 g lactulose and 2 g mannitol. For the next 6 h, participants were allowed to eat, except for specified foods (milk and dairy products, simple sugars, high doses of vitamin C and mannitol), and were asked to collect all urine passed into one container. Finally, 400-μL aliquots of urine from the collection container were assayed. These 400-μL urine aliquots were mixed with 40 μL of an internal standard (myo-inositol, 20 mg/mL) and evaporated to dryness by lyophilization. Next, 200 μL of anhydrous pyridine in hydroxylamine (25 mg/mL) was added, mixed, and heated to 70 °C for one hour. The sample was then centrifuged at 800× *g* for 5 min, and 200 μL of supernatant was collected. Next, sugars were silylated with 100 µL of N-trimethylsilylimidazole for 30 min at 70 °C and assayed by gas chromatography. Gas chromatography was performed with an Agilent Technologies 7890A GC System and capillary column (15 m × 0.530 mm, 1.50 μm; Supelco, Bellefonte, PA, USA). Chromatographic conditions included an initial temperature of 220 °C for 5 min, increased at a rate of 10 °C/min for 2 min, 5 °C/min for 4 min, and 3.5 °C/min for 4 min to a final temperature of 274 °C, which was maintained for 7 min. The total time was approximately 22 min, and hydrogen was the carrier gas. Lactulose, mannitol, and myo-inositol were identified by comparing their retention times with those of commercially available standards.

#### 4.2.2. Stool Concentrations of Zonulin, Calprotectin and Beta-Defensin-2 Assays

The concentration of zonulin, calprotectin and beta-defensin-2 in stool were determined with a competitive enzyme-linked immunosorbent assay (ELISA) kit (Immundiagnostik AG, Bensheim, Germany) following the manufacturer’s protocol. Assay plate wells from the kits were coated with polyclonal antibodies; zonulin in the stool samples was conjugated to a biotinylated zonulin tracer and then immobilized on the plate. Calprotectin and beta-defensin-2 in the stool samples were conjugated to a peroxidase labelled conjugate. Tetramethylbenzidine (TMB) was used as a substrate for peroxidase. Finally, an acidic stop solution was added to terminate the reaction. The absorbance was measured by a photometer at 450 nm. A dose-response curve of the absorbance unit (optical density, OD at 450 nm) vs. concentration was generated using the values obtained from the standard [41,42,43]. Due to the lack of compliance in stool collection, zonulin concentration was evaluated in 32 patients, calprotectin in 31 patients, and beta-defensin-2 in 14 patients.

#### 4.2.3. Reference Limits

We have established internal upper reference limits of the lactulose to mannitol ratio (LMR) as <0.035, referring to previous studies and the group experience [40,44]. Calprotectin levels in stool <50 μg/mL were regarded as normal, 50–100 μg/mL as borderline positive and >100 μg/mL as positive [45]. For zonulin stool concentrations, the normal range is <30 ng/mL, referring to previous studies and our own experience [44]. For beta-defensin-2, the normal range is considered to be 8–60 ng/mL [42].

#### 4.2.4. Statistical Analysis

Results were presented as mean with standard deviation or as median with interquartile range (IQR) or extreme values regarding fulfillment criteria of normal distribution. Categorical variables were presented as numbers or numbers with the occurrence. The normality of investigated variables was checked with the Shapiro-Wilk test. For correlation assessment, the Spearman test or point-biserial correlation was performed. Differences between variables were analyzed with a T-test for parametric data, otherwise with a U-Mann-Whitney or Wilcoxon test in view of the dependency of variables. Differences in categorical data were analyzed with Chi2 or Fisher’s exact test if the number of observations was low. To compare more than two nonparametric dependent variables, a Friedman’s ANOVA test was used. Missing data were not substituted, but data available for each analysis were used. Two-tailed *p* < 0.05 was considered significant. For statistical analysis, Statistica 12 software (StatSoft Polska, Cracow, Poland) was used.

## 5. Conclusions

Most patients undergoing allo-HCT had a disrupted intestinal barrier and increased intestinal permeability measured as LMR after SAT before the start of the conditioning therapy. HCT conditioning caused further damage to the gut epithelium and led to the increase in intestinal permeability measured as ΔLMR. We did not find any association between permeability assay and zonulin, calprotectin or beta-defensin-2 concentrations. We confirmed the role of faecal calprotectin as a marker for aGVHD development. The role of zonulin and beta-defensin-2 in increased intestinal permeability after allo-HCT requires further research.

## Figures and Tables

**Table 1:** Associations between median intestinal permeability (LMR) on day −7 before transplantation, day +7 after transplantations, and median difference between these values (ΔLMR) and characteristics of patients and transplantation. A *p*-value less than 0.05 is considered to be statistically significant.

Patient or Transplant Characteristic	N	LMR Day −7, Median (IQR)	*p*	LMR Day +7 Median (IQR)	*p*	ΔLMR Median (IQR)	*p*
All	45	0.04 (0.23)		0.16 (0.57)		0.02 (0.18)	x
Increased LMR on day −7	24	0.19	<0.001	0.31	<0.001	0.06	0.41
Normal LMR on day −7	21	0.028		0.038		0.016	
Sex							
woman	22	0.040 (0.041)	0.955	0.136 (0.588)	0.865	0.025 (0.152)	0.665
men	23	0.043 (0.302)		0.271 (0.574)		0.020 (0.196)	
type of conditioning							
RIC	13	0.040 (0.038)	0.853	0.161 (0.628)	0.528	0.036 (0.280)	0.665
myeloablative	32	0.040 (0.243)		0.155 (0.527)		0.020 (0.139)	
age							
<65 years old	40	0.040 (0.067)	0.367	0.108 (0.525)	0.164	0.025 (0.185)	0.639
≥65 years old	5	0.026 (0.999)		0.700 (0.783)		0.020 (0.071)	
HCT-CI							
≤2	25	0.041 (0.041)	0.035	0.161 (0.301)	0.364	0.036 (0.190)	0.903
>2	6	0.430 (0.821)		0.670 (0.926)		0.090 (0.185)	
WHO							
0	28	0.037 (0.071)	0.423	0.181 (0.583)	0.737	0.028 (0.235)	0.423
>0	17	0.044 (0.350)		0.110 (0.551)		0.018 (0.065)	

**Table 2:** Intestinal permeability expressed as LMR and stool biomarkers concentrations before the conditioning and in the post-transplant period. Normal value for LMR is <0.035, for zonulin it is <30 ng/mL, for calprotectin it is <50 μg/mL, and for beta-defensin-2 it is 8–60 ng/mL. A *p*-value less than 0.05 is considered to be statistically significant.

Parameter	−7	+7	+14	+21	*p* (−7 vs. +7)	*p* (−7:+21)
	Median (IQR)	Median (IQR)	Median (IQR)	Median (IQR)		
LMR	0.04 (0.23)	0.16 (0.57)			<0.001	-
Zonulin [ng/mL]	76.45 (157.2)	39.95 (75.1)	43.00 (47.75)	41.45 (54.4)	<0.001	<0.001
Calprotectin [μg/mL]	15.50 (25.2)	21.50 (57.9)	19.30 (44.9)	16.90 (35.5)	0.374	0.451
Beta-defensin-2 [ng/mL]	17.90 (22.8)	7.80 (6.8)	4.31 (11.0)	6.20 (6.8)	0.101	0.006

**Table 3:** Calprotectin and zonulin stool concentrations before the conditioning and in the post-transplant period in patients with and without aGVHD, * *p* < 0.05 between −7 and +7. Normal values for zonulin is <30 ng/mL and for calprotectin it is <50 μg/mL. A *p*-value less than 0.05 is considered to be statistically significant.

Parameter	−7	7	14	21	*p*
	Median (IQR)	Median (IQR)	Median (IQR)	Median (IQR)	
aGVHD					
Calprotectin [μg/mL]	10.3 (24.2)	64.8 (167.4)	22.1 (26.0)	29.7 (40.3)	*p* > 0.05 *
Zonulin [ng/mL]	51.4 (31.9)	42.8 (104.7)	35.5 (21.0)	30.6 (116.4)	*p* > 0.05
without aGVHD					
Calprotectin [μg/mL]	15.6 (22.6)	14.1 (39.8)	17.7 (45.9)	14.9 (35.0)	*p* > 0.05
Zonulin [ng/mL]	86.7 (158.4)	40 (63.1)	45.2 (52.8)	47.5 (51.4)	*p* > 0.05

**Table 4:** Patients characteristics (n = 51).

Age in years, median (range)	54.0 (19–67)
Male sex, n (%)	23 (45)
Performance Status, n (%)	
0	30 (59)
1	21 (41)
Conditioning treatment, n (%)	
myeloablative	37 (73)
RIC	14 (27)
GVHD prophylaxis regimen, n (%)	
CSA/MTX	39 (76)
other	12 (24)
Disease, n (%)	
Acute leukemia	32 (63)
Aplastic anemia	3 (6)
Myelodysplastic syndrome	6 (12)
Chronic myeloid leukemia	3 (6)
Others *	7 (13)
Disease status before transplantation, n (%)	
CR	28 (54)
CR, MRD(+)	9 (18)
PR	5 (10)
PD	2 (4)
Chronic phase	2 (4)
Transplantation upfront	5 (10)
Complications, n (%):	
infections within 30 days (included FN):	30 (58)
confirmed bacterial infection	15 (29)
aGvHD	16 (31)
aGvHD grade III and IV	6 (12)
mucositis	36 (71)
mucositis grade III and IV	22 (43)

Note. Data are median or number of patients. * myelofibrosis (n = 2), MonoMac Syndrome n = 1, Blastic plasmacytoid dendritic cell neoplasm n = 1, Chronic Lymphocytic leukemia n = 1, Chronic myelomonocytic leukemia n = 2. Abbreviations: FN (febrile neutropenia), CsA (cyclosporine A), MTX (metothrexate), RIC (reduced intensity conditioning), aGvHD (acute graft versus host disease), CR (complete remission), MRD + (molecular residual disease), PR (partial remission), PD (progressive disease).

## Data Availability

Not applicable.

## Supplementary Materials

The following supporting information can be downloaded at: https://www.mdpi.com/article/10.3390/ijms232415962/s1.

## References

[1] Jenq R.R., Ubeda C., Taur Y., Menezes C.C., Khanin R., Dudakov J., Liu C., West M.L., Singer N.V., Equinda M.J. (2012). Regulation of intestinal inflammation by microbiota following allogeneic bone marrow transplantation. J. Exp. Med..

[2] Taur Y., Xavier J., Lipuma L., Ubeda C., Goldberg J., Gobourne A., Lee Y.J., Dubin K.A., Socci N.D., Viale A. (2012). Intestinal Domination and the Risk of Bacteremia in Patients Undergoing Allogeneic Hematopoietic Stem Cell Transplantation. Clin. Infect. Dis..

[3] Jenq R.R., Taur Y., Devlin S.M., Ponce D.M., Goldberg J.D., Ahr K.F., Littmann E.R., Ling L., Gobourne A.C., Miller L.C. (2015). Intestinal Blautia Is Associated with Reduced Death from Graft-versus-Host Disease. Biol. Blood Marrow Transplant..

[4] Holler E., Butzhammer P., Schmid K., Hundsrucker C., Koestler J., Peter K., Zhu W., Sporrer D., Hehlgans T., Kreutz M. (2014). Metagenomic Analysis of the Stool Microbiome in Patients Receiving Allogeneic Stem Cell Transplantation: Loss of Diversity Is Associated with Use of Systemic Antibiotics and More Pronounced in Gastrointestinal Graft-versus-Host Disease. Biol. Blood Marrow Transplant..

[5] Shono Y., Docampo M.D., Peled J.U., Perobelli S.M., Velardi E., Tsai J.J., Slingerland A.E., Smith O.M., Young L.F., Gupta J. (2016). Increased GVHD-related mortality with broad-spectrum antibiotic use after allogeneic hematopoietic stem cell transplantation in human patients and mice. Sci. Transl. Med..

[6] Mathewson N.D., Jenq R., Mathew A.V., Koenigsknecht M., Hanash A., Toubai T., Oravecz-Wilson K., Wu S.-R., Sun Y., Rossi C. (2016). Gut microbiome–derived metabolites modulate intestinal epithelial cell damage and mitigate graft-versus-host disease. Nat. Immunol..

[7] Harris B., Morjaria S.M., Littmann E.R., Geyer A.I., Stover D.E., Barker J.N., Giralt S.A., Taur Y., Pamer E.G. (2016). Gut Microbiota Predict Pulmonary Infiltrates after Allogeneic Hematopoietic Cell Transplantation. Am. J. Respir. Crit. Care Med..

[8] Taur Y., Jenq R., Perales M.-A., Littmann E.R., Morjaria S., Ling L., No D., Gobourne A., Viale A., Dahi P. (2014). The effects of intestinal tract bacterial diversity on mortality following allogeneic hematopoietic stem cell transplantation. Blood.

[9] Menard S., Cerf-Bensussan N., Heyman M.B. (2010). Multiple facets of intestinal permeability and epithelial handling of dietary antigens. Mucosal Immunol..

[10] Lalla R.V., Sonis S.T., Peterson D.E. (2008). Management of Oral Mucositis in Patients Who Have Cancer. Dent. Clin. N. Am..

[11] Johansson J.-E., Ekman T. (1997). Gastro-intestinal toxicity related to bone marrow transplantation: Disruption of the intestinal barrier precedes clinical findings. Bone Marrow Transplant..

[12] Johansson J.E., Brune M., Ekman T. (2001). The gut mucosa barrier is preserved during allogeneic, haemopoietic stem cell trans-plantation with reduced intensity conditioning. Bone Marrow Transplant..

[13] Blijlevens N.M.A., Donnelly J.P., M’Rabet L., De Pauw B.E., Land B.V. (2004). Measuring mucosal damage induced by cytotoxic therapy. Support. Care Cancer.

[14] Johansson J.-E., Ekman T. (2007). Gut Toxicity During Hemopoietic Stem Cell Transplantation May Predict Acute Graft-Versus-Host Disease Severity in Patients. Am. J. Dig. Dis..

[15] Cooke K.R., Gerbitz A., Crawford J.M., Teshima T., Hill G.R., Tesolin A., Rossignol D.P., Ferrara J.L. (2001). LPS antagonism reduces graft-versus-host disease and preserves graft-versus-leukemia activity after experimental bone marrow transplantation. J. Clin. Investig..

[16] Peled J.U., Gomes A.L., Devlin S.M., Littmann E.R., Taur Y., Sung A.D., Weber D., Hashimoto D., Slingerland A.E., Slingerland J.B. (2020). Microbiota as Predictor of Mortality in Allogeneic Hematopoietic-Cell Transplantation. N. Engl. J. Med..

[17] Bates D.W., Parsonnet J., Ketchum P.A., Miller E.B., Novitsky T.J., Sands K., Hibberd P.L., Graman P.S., Lanken P.N., Schwartz J.S. (1998). Limulus Amebocyte Lysate Assay for Detection of Endotoxin in Patients with Sepsis Syndrome. Clin. Infect. Dis..

[18] Strutz F., Heller G., Krasemann K., Krone B., Müller G.A. (1999). Relationship of antibodies to endotoxin core to mortality in medical patients with sepsis syndrome. Intensiv. Care Med..

[19] Blijlevens N.M.A., Lutgens L.C.H.W., Schattenberg A.V.M.B., Donnelly J.P. (2004). Citrulline: A potentially simple quantitative marker of intestinal epithelial damage following myeloablative therapy. Bone Marrow Transplant..

[20] Crenn P., Coudray–Lucas C., Thuillier F., Cynober L., Messing B. (2000). Postabsorptive plasma citrulline concentration is a marker of absorptive enterocyte mass and intestinal failure in humans. Gastroenterology.

[21] Vreugdenhil A.C., Wolters V.M., Adriaanse M.P., Van den Neucker A.M., van Bijnen A.A., Houwen R., Buurman W.A. (2011). Additional value of serum I-FABP levels for evaluating celiac disease activity in children. Scand. J. Gastroenterol..

[22] Prasad S., Mingrino R., Kaukinen K., Hayes K.L., Powell R.M., Macdonald T.T., Collins J. (2005). Inflammatory processes have differential effects on claudins 2, 3 and 4 in colonic epithelial cells. Lab. Investig..

[23] Silberer H., Küppers B., Mickisch O., Baniewicz W., Drescher M., Traber L., Kempf A., Schmidt-Gayk H. (2005). Fecal leukocyte proteins in inflammatory bowel disease and irritable bowel syndrome. Clin. Lab..

[24] Aasebo A.T., Gedde-Dahl T., Reims H.M., Baekkevold E.S., Jahnsen F.L. (2022). Calprotectin Expressing Donor-Derived Macro-phages Increase in Acute Gastrointestinal Graft-Versus-Host Disease. Transplant. Cell. Ther..

[25] Fasano A. (2008). Physiological, Pathological, and Therapeutic Implications of Zonulin-Mediated Intestinal Barrier Modulation: Living Life on the Edge of the Wall. Am. J. Pathol..

[26] Wang W., Uzzau S., Goldblum S.E., Fasano A. (2000). Human zonulin, a potential modulator of intestinal tight junctions. J. Cell. Sci..

[27] Fasano A. (2012). Intestinal Permeability and Its Regulation by Zonulin: Diagnostic and Therapeutic Implications. Clin. Gastroenterol. Hepatol..

[28] Fusco A., Savio V., Donniacuo M., Perfetto B., Donnarumma G. (2021). Antimicrobial Peptides Human Beta-Defensin-2 and -3 Protect the Gut During Candida albicans Infections Enhancing the Intestinal Barrier Integrity: In Vitro Study. Front. Cell. Infect. Microbiol..

[29] Han F., Zhang H., Xia X., Xiong H., Song D., Zong X., Wang Y. (2015). Porcine β-Defensin 2 Attenuates Inflammation and Mucosal Lesions in Dextran Sodium Sulfate–Induced Colitis. J. Immunol..

[30] Costa-Lima C., De Paula E.V. (2014). ‘Leaky gut’ in hematological malignancies. Rev. Bras. De Hematol. E Hemoter..

[31] Sundström G.M., Wahlin A., Nordin-Andersson I., Suhr O.B. (1998). Intestinal permeability in patients with acute myeloid leu-kemia. Eur. J. Haematol..

[32] Leite J.B., Vilela E.G., Torres H.O.D.G., Ferrari M.D.L.D.A., da Cunha A.S. (2014). Intestinal permeability in leukemic patients prior to chemotherapy. Rev. Bras. Hematol. Hemoter..

[33] Nocerino R., De Filippis F., Cecere G., Marino A., Micillo M., Di Scala C., de Caro C., Calignano A., Bruno C., Paparo L. (2020). The therapeutic efficacy of Bifidobacterium animalis subsp. lactis BB-12(^®^) in infant colic: A randomised, double blind, placebo-controlled trial. Aliment. Pharmacol. Ther..

[34] Siljander H., Jason E., Ruohtula T., Selvenius J., Koivusaari K., Salonen M., Ahonen S., Honkanen J., Ilonen J., Vaarala O. (2021). Effect of Early Feeding on Intestinal Permeability and Inflammation Markers in Infants with Genetic Susceptibility to Type 1 Diabetes: A Randomized Clinical Trial. J. Pediatr..

[35] Savilahti E.M., Kukkonen A.K., Tuure T., Kuitunen M., Haahtela T. (2011). Intestinal defensin secretion in infancy is associated with the emergence of sensitization and atopic dermatitis. Clin. Exp. Allergy.

[36] Nocerino R., Paparo L., Terrin G., Pezzella V., Amoroso A., Cosenza L., Cecere G., De Marco G., Micillo M., Albano F. (2017). Cow’s milk and rice fermented with Lactobacillus paracasei CBA L74 prevent infectious diseases in children: A randomized controlled trial. Clin. Nutr..

[37] Malik M.N., Rafae A., Durer C., Durer S., Anwer F. (2019). Fecal Calprotectin as a Diagnostic and Prognostic Biomarker for Gas-trointestinal Graft Versus Host Disease: A Systematic Review of Literature. Cureus.

[38] Legert K.G., Remberger M., Ringdén O., Heimdahl A., Dahllöf G. (2014). Reduced intensity conditioning and oral care measures prevent oral mucositis and reduces days of hospitalization in allogeneic stem cell transplantation recipients. Support. Care Cancer.

[39] Haverman T.M., Raber-Durlacher J.E., Rademacher W.M.H., Vokurka S., Epstein J.B., Huisman C., Hazenberg M., De Soet J.J., De Lange J., Rozema F.R. (2014). Oral Complications in Hematopoietic Stem Cell Recipients: The Role of Inflammation. Mediat. Inflamm..

[40] Bjarnason I., Macpherson A., Hollander D. (1995). Intestinal permeability: An overview. Gastroenterology.

[41] Zonulin Elisa. https://www.immundiagnostik.com/media/pages/testkits/k-5600/17c79275c91653271285/k5600_2022-02-16_idk-zonulin-elisa.pdf.

[42] ß-Defensin 2 ELISA. https://www.immundiagnostik.com/media/pages/testkits/k-6500/facd9e2d16-1653271285/k6500_2022-02-24_beta-defensin.pdf.

[43] Calprotectin Elisa. https://www.idkna.com/docs/KR6927_2022-02-15_IDK_Calprotectin_Stuhl_1h.pdf.

[44] Hałasa M., Maciejewska D., Ryterska K., Baśkiewicz-Hałasa M., Safranow K., Stachowska E. (2019). Assessing the Association of Elevated Zonulin Concentration in Stool with Increased Intestinal Permeability in Active Professional Athletes. Medicina.

[45] Bjarnason I. (2017). The Use of Fecal Calprotectin in Inflammatory Bowel Disease. Gastroenterol. Hepatol..

