# Supplementary material for: Increased Intestinal Permeability and Stool Zonulin, Calprotectin and Beta-Defensin-2 Concentrations in Allogenic Hematopoietic Cell Transplantation Recipients
Source: Int J Mol Sci. 2022 Dec 15;23(24):15962. doi: 10.3390/ijms232415962 (PMC9781277; doi:10.3390/ijms232415962)
Supplement: Supplementary file 1 [file ijms-23-15962-s001.zip › ijms-2055808-supplementary.pdf]

	Infection (median, IQR)	without infection (median, IQR)	p
LMR			
day -7	0.206 (0.177)	0.039 (0.167)	0.914
day +7	0.697 (0.643)	0.110 (0.531)	0.224
ΔLMR	0.284 (0.268)	0.017 (0.142)	0.090
Zonulin (ng/ml)			
day -7	112.150 (73.700)	83.800 (185.650)	0.315
day +7	55.550 (27.212)	51.000 (97.800)	0.370
day +14	75.237 (41.837)	43.425 (46.186)	0.579
day +21	80.787 (43.687)	34.750 (51.813)	0.648
Calprotectin (ug/ml)			
day -7	19.352 (11.142)	16.617 (27.767)	0.470
day +7	111.447 (106.510)	24.649 (39.087)	0.716
day +14	43.073 (39.385)	22.617 (38.011)	0.842
day +21	59.952 (54.660)	19.830 (32.809)	0.601
Beta-defensin-2 (ng/ml)			
day -7	49.050 (40.250)	17.900 (15.850)	0.456
day +7	6.910 (1.494)	8.800 (9.800)	0.463
day +14	14.250 (14.250)	4.310 (10.600)	0.947
day +21	14.050 (7.050)	5.200 (3.400)	0.242

Supplemental Table S1 Median values of analyzed parameter for group with confirmed bacterial infection within 30 days after allo-HCT and group without this complication. Normal values; for LMR is <0.035, for zonulin is<30ng/ml, for calprotectin is <50ug/ml, for beta-defensin-2 is 8-60 ng/ml. A p-value less than 0.05 is considered to be statistically significant

	aGVHD (median, IQR)	without aGVHD	p
LMR			
day -7	0.037 (0.078)	0.040 (0.237)	0.242
day +7	0.332 (0.569)	0.091 (0.528)	0.576
ΔLMR	0.085 (0,281)	0.018 (0.118)	0.149
Zonulin (ng/ml)			
day -7	51.425 (37.875)	86.725 (158.350)	0.552
day +7	42.775 (104.687)	39.950 (63.100)	0.193
day +14	35.500 (21.000)	45.200 (52.825)	0.214
day +21	30.600 (116.412)	47.500 (51.400)	0.189
Calprotectin (ug/ml)			
day -7	10.320 (24.188)	15.613 (22.623)	0.547
day +7	64.758 (167.403)	14.060 (39.820)	0.040
day +14	22.128 (26,007)	17.666 (45.943)	0.319
day +21	29.738 (40.252)	16.859 (35.033)	0.085
Beta-defensin-2 (ng/ml)			
day -7	29.79 (2.220)	23.200 (19.450)	0.055
day +7	38.300 (34.7)	7.800 (4.789)	0.693
day +14	n/a	6.020 (10.600)	n/a
day +21	7.500 (5.7)	6.200 (5.100)	0.923

Supplemental Table S2 Median values of analyzed parameter for group with aGVHD and group without this complication. Normal values; for LMR is <0.035, for zonulin is<30ng/ml, for calprotectin is <50ug/ml, for beta-defensin-2 is 8-60 ng/ml , n/a; not applicable (no beta-defensin-2 data in aGvGD group). A p-value less than 0.05 is considered to be statistically significant

	mucositis (median, IQR)	without mucositis (median, IQR)	p
LMR			
day -7	0.039 (0.100)	0.042 (0.259)	0.109
day +7	0.271 (0.653)	0.110 (0.531)	0.576
ΔLMR	0.020 (0.445)	0.020 (0.141)	0.201
Zonulin (ng/ml)			
day -7	75.550 (99.187)	83.800 (154.350)	0.340
day +7	34.550 (111.200)	47.425 (64.412)	0.616
day +14	41.125 (44.450)	43.000 (48.585)	0.048
day +21	49.800 (139.150)	34.100 (47.300)	0.284
Calprotectin (ug/ml)			
day -7	17.497 (24.188)	13.580 (22.787)	0.489
day +7	25.877 (52.798)	15.866 (47.944)	0.413
day +14	16.501 (24.066)	27.201 (109.550)	0.598
day +21	13.440 (33.200)	19.088 (44.431)	0.074
Beta-defensin-2 (ng/ml)			
day -7	15.700 (17.950)	25.00 (20.950)	0.361
day +7	8.800 (15.000)	7.800 (4.289)	0.255
day +14	4.310 (5.865)	7.900 (11.850)	0.457
day +21	6.200 (11.400)	6.100 (4.250)	0.928

Supplemental Table S3 Median values of analyzed parameter for group with mucositis of grade III and IV and group without this complication. Normal values; for LMR is <0.035, for zonulin is<30ng/ml, for calprotectin is <50ug/ml, for beta-defensin-2 is 8-60 ng/ml. A p-value less than 0.05 is considered to be statistically significant
